# Tumor Burden in Patients With Hepatocellular Carcinoma Undergoing Transarterial Chemoembolization: Head-to-Head Comparison of Current Scoring Systems

**DOI:** 10.3389/fonc.2022.850454

**Published:** 2022-02-23

**Authors:** Lukas Müller, Felix Hahn, Timo Alexander Auer, Uli Fehrenbach, Bernhard Gebauer, Johannes Haubold, Sebastian Zensen, Moon-Sung Kim, Michel Eisenblätter, Thierno D. Diallo, Dominik Bettinger, Verena Steinle, De-Hua Chang, David Zopfs, Daniel Pinto dos Santos, Roman Kloeckner

**Affiliations:** ^1^ Department of Diagnostic and Interventional Radiology, University Medical Center of the Johannes Gutenberg University Mainz, Mainz, Germany; ^2^ Department of Radiology, Charité – University Medicine Berlin, Berlin, Germany; ^3^ Department of Diagnostic and Interventional Radiology and Neuroradiology, University Hospital Essen, Essen, Germany; ^4^ Department of Diagnostic and Interventional Radiology, Freiburg University Hospital, Freiburg, Germany; ^5^ Department of Medicine II, Medical Center University of Freiburg, Faculty of Medicine, University of Freiburg, Freiburg, Germany; ^6^ Department of Diagnostic and Interventional Radiology, University Medical Center Heidelberg, Heidelberg, Germany; ^7^ Department of Radiology, University Hospital Cologne, Cologne, Germany

**Keywords:** hepatocellular carcinoma, risk scoring, prognosis prediction, tumor burden, transarterial chemoembolization

## Abstract

**Objectives:**

Recently, several scoring systems for prognosis prediction based on tumor burden have been promoted for patients with hepatocellular carcinoma (HCC) undergoing transarterial chemoembolization (TACE). This multicenter study aimed to perform the first head-to-head comparison of three scoring systems.

**Methods:**

We retrospectively enrolled 849 treatment-naïve patients with HCC undergoing TACE at six tertiary care centers between 2010 and 2020. The tumor burden score (TBS), the Six-and-Twelve score (SAT), and the Seven-Eleven criteria (SEC) were calculated based on the maximum lesion size and the number of tumor nodes. All scores were compared in univariate and multivariate regression analyses, adjusted for established risk factors.

**Results:**

The median overall survival (OS) times were 33.0, 18.3, and 12.8 months for patients with low, medium, and high TBS, respectively (p<0.001). The median OS times were 30.0, 16.9, and 10.2 months for patients with low, medium, and high SAT, respectively (p<0.001). The median OS times were 27.0, 16.7, and 10.5 for patients with low, medium, and high SEC, respectively (p<0.001). In a multivariate analysis, only the SAT remained an independent prognostic factor. The C-Indexes were 0.54 for the TBS, 0.59 for the SAT, and 0.58 for the SEC.

**Conclusion:**

In a direct head-to-head comparison, the SAT was superior to the TBS and SEC in survival stratification and predictive ability. Therefore, the SAT can be considered when estimating the tumor burden. However, all three scores showed only moderate predictive power. Therefore, tumor burden should only be one component among many in treatment decision making.

## Introduction

Hepatocellular carcinoma (HCC) is the fifth most common cancer entity worldwide and is responsible for the second-highest number of cancer-related deaths (1, 2). Following the current guidelines from the European Association for the Study of the Liver (EASL) and the American Association for the Study of Liver Diseases (AASLD), the Barcelona Clinic Liver Cancer (BCLC) classification system is the preferred framework for treatment allocations and prognosis predictions (3, 4). According to the BCLC classification, transarterial chemoembolization (TACE) is the standard of care for patients with intermediate-stage HCC (5, 6). However, there is substantial heterogeneity among patients with intermediate-stage HCC, due to considerable differences in tumor burden and liver function (7). This heterogeneity hampers prognosis prediction, and consequently, it remains difficult to make treatment decisions for these patients. To support clinicians in the decision-making process, several scoring systems have been proposed (8–10). However, all have failed in external validation (11, 12).

Recently, a novel scoring system, based only on tumor burden, was promoted by Wang et al. (13). They called this system the Six-and-Twelve (SAT) score. It sums the number of tumor nodes and tumor size to obtain a readily applicable stratification system. In the original study, the score showed promising performance, but in an external validation, contrary results were found for the prognosis prediction (14–18).

Additionally, the Tumor Burden Score (TBS) is a novel scoring system, based on a combination of tumor number and tumor size. The TBS was originally developed for patients undergoing resections of colorectal liver metastasis. However, very recently, the TBS was successfully applied to stratify patients with HCC undergoing TACE (19).

A third novel scoring system, the Seven-Eleven criteria (SEC), was recently developed to estimate tumor burden (20). These criteria also include tumor size and the number of tumor nodes in the calculation. The introduction of the SEC aimed at improving the already existing up-to-7 criteria, which have been originally developed for patients with HCC undergoing liver transplantation, and the up-to-11 criteria, which are part of the BCLC stage B subclassification, through a combination of both (21–23).

Overall, it remains unclear which of these tools might be optimal for estimating tumor burden in HCC. A direct head-to-head comparison of all scoring systems is lacking. Furthermore, it is questionable whether tumor burden alone is sufficient to support clear-cut treatment decisions, because HCC development is associated with liver cirrhosis in more than 80% of Western patients (3). Thus, most patients have two diseases: HCC and liver cirrhosis, and both contribute to an impaired prognosis. The complex interactions between cirrhosis and tumor development and progression might be underrepresented in a scoring system based only on estimates of the actual tumor burden.

This study aimed to perform the first head-to-head comparison of SAT, TBS, and SEC performances in predicting HCC prognosis. Additionally, we aimed to compare these scores to several established scoring systems in terms of prognostic power.

## Materials and Methods

This multicentric retrospective study was approved by the Ethics committee of the Medical Association of Rhineland Palatinate, Mainz, Germany (permit number 2021-15913). The other responsible Ethics committees followed this approval. The requirement for informed consent was waived, due to the retrospective nature of the analysis. Patient records and information were anonymized and de-identified prior to analysis. TRIPOD guidelines were applied to the organization of this manuscript (24).

### Patients

A total of six German tertiary care centers participated. Patient inclusion criteria were: (1) TACE performed between January 2010 and December 2020; (2) age >18 years; (3) a histological- or image-derived HCC diagnosis, based on EASL criteria; (4) no treatment performed prior to TACE; (5) no liver transplantation or tumor resection performed during the follow-up period after TACE; and (6) computed tomography (CT) or magnetic resonance imaging (MRI) performed prior to treatment initiation to assess tumor number and tumor sizes in full detail. The exclusion criteria were: (1) age <18 years; (2) any treatment performed prior to TACE; (3) liver transplantation or tumor resection performed during the follow-up period after TACE; and (4) missing computed tomography (CT) or magnetic resonance imaging (MRI) prior to treatment initiation or insufficient image quality.

### Diagnosis, Treatment, and Follow-Up

HCC was diagnosed either non-invasively, based on image-derived EASL criteria or a histological biopsy assessment (3, 25). All patients underwent contrast-enhanced CT or MRI prior to the first TACE for precise estimation of tumor burden and for procedure planning. Prior to each treatment cycle, each case was repeatedly discussed by an interdisciplinary tumor board, which included hepatologists/oncologists, diagnostic and interventional radiologists, visceral/transplant surgeons, pathologists, and radiation therapists. TACE was performed in a standardized manner, as previously described (26–28). Follow-up examinations included cross-sectional imaging, a clinical examination, and a blood sample analysis (25). The primary endpoint was the median overall survival (OS), defined as the duration between the initial TACE session and death. Patients that had not died at the end of study or were lost to follow-up were censored at the last available contact date. We extracted data on patient demographics, liver disease status and etiology, laboratory parameters, and TACE-related parameters from the hospital information system and the laboratory database at each center. We accessed tumor burden information, including the tumor growth pattern, number of lesions, and the diameter of the largest target lesion, from the radiology information system and the picture archiving and communication system.

### Scoring Systems

The SAT, TBS, and SEC were calculated as described in the original publications (13, 19, 20) (**Table 1**). We also determined the BCLC grade, the albumin-bilirubin (ALBI) grade, the Child-Pugh grade, the hepatoma arterial-embolization prognostic (HAP) score, and the modified HAP (mHAP-II) score, as previously reported (6, 9, 29–31).

**Table 1:** Calculations of tumor burden scores.

Scoring system	Calculation	Risk group		
		Low	Medium	High
Tumor Burden Score (TBS)	TBS = square root [(maximum tumor diameter)^2^ + (number of tumors)^2^]	<3.36	3.36 – 13.74	>13.74
Six-and-Twelve Score (SAT)	SAT = the largest diameter (cm) + tumor number	≤6	>6 but ≤12	>12
Seven-Eleven Criteria (SEC)	SEC = the largest diameter (cm) + tumor number	≤7	>7 but ≤11	>11

### Statistical Analysis

All statistical analyses and graphic designs were performed with R 4.0.3 (A Language and Environment for Statistical Computing, R Foundation for Statistical Computing, http://www.R-project.org; last accessed 30 November 2021). Categorical and binary baseline parameters are reported as absolute numbers and percentages. Continuous data are reported as the median and range. Standardized cut-offs for the laboratory parameters were derived from our laboratory database. Cut-off values for TBS, SAT and SEC were adopted from the original publications (13, 19, 20). The distributions of patients, according to TBS SAT and SEC, were compared with the Chi-Square test for categorial variables. The packages “survminer” and “survival” (https://cran.r-project.org/package=survminer, https://CRAN.R-project.org/package=survival, last accessed 30 November 2021) were used to perform survival analyses and for drawing the Kaplan-Meier curves. Strata comparisons were evaluated with log-rank tests. Multivariate Cox proportional hazards regression models were built to assess hazard ratios (HRs) and corresponding 95% confidence intervals (CIs) to determine the effect of the risk stratification and to evaluate the roles of included factors. Harrell’s C concordance index (C-Index) was calculated with the “Hmisc” package (https://cran.r-project.org/package=Hmisc, last accessed 30 November 2021). A C-Index of 0.5 indicated no predictive ability, and a C-Index of 1.0 indicated perfect predictive power (32). Prediction error curves were based on the Brier score (package “pec,” https://cran.r-project.org/package=pec, accessed October 2021). The Brier score was evaluated at specific timepoints, and it was defined as the mean squared difference between the observed outcome and the predicted outcome probability (33). The prediction error was summarized by calculating the integrated Brier score (IBS) over the study interval [0 months to 60 months]. P-values <0.05 were considered significant for all tests.

## Results

### Baseline Characteristics

The analyses included 849 patients that met the full set of inclusion and exclusion criteria. The baseline characteristics of all patients are provided in **Table 2**. A subgroup analysis was performed on 418 (49.2%) patients within the intermediate stage (BCLC stage B), for whom TACE treatment is the recommended first-line therapy according to current Western guidelines (3, 4).

**Table 2:** Baseline characteristics of patients with HCC undergoing TACE.

Characteristic		All patients (n = 849)
Age, years		67 (60 – 74)
Sex	
Female		163 (19.2)
Male		686 (80.8)
Etiology	
Alcohol		330 (38.9)
Viral		261 (30.7)
Other		172 (20.3)
No cirrhosis		86 (10.1)
Child-Pugh stage	
No cirrhosis*		86 (10.1)
A		447 (52.7)
B		262 (30.9)
C		54 (6.3)
BCLC stage	
0		15 (1.8)
A		269 (31.7)
B		418 (49.2)
C		121 (14.3)
D		26 (3.0)
Max. tumor size, cm		4.0 (2.7 – 6.0)
Tumor number	
Unifocal		321 (37.8)
Multifocal		528 (62.2)
Albumin level, g/l		35 (30 – 40)
Bilirubin level, mg/dl		1.1 (0.7 – 1.9)
Platelet count, platelets/nl		121 (81 – 187)
AST level, U/l		60 (42 – 89)
ALT level, U/l		40 (27 – 61)
INR		1.1 (1.1 – 1.3)
AFP level, ng/ml		16.3 (5.4 – 237.1)
Type of TACE		
Conventional TACE		422 (49.7)
Drug-eluting beads TACE		427 (50.3)

^*^formally within the Child Pugh stage A. Values are the number (%) or the median (range), as indicated. BCLC, Barcelona Clinic Liver Cancer; AST, aspartate aminotransferase; ALT, alanine aminotransferase; AFP, alpha-fetoprotein.

### Distribution and Comparison of Tumor Burden

**Figure 1:**
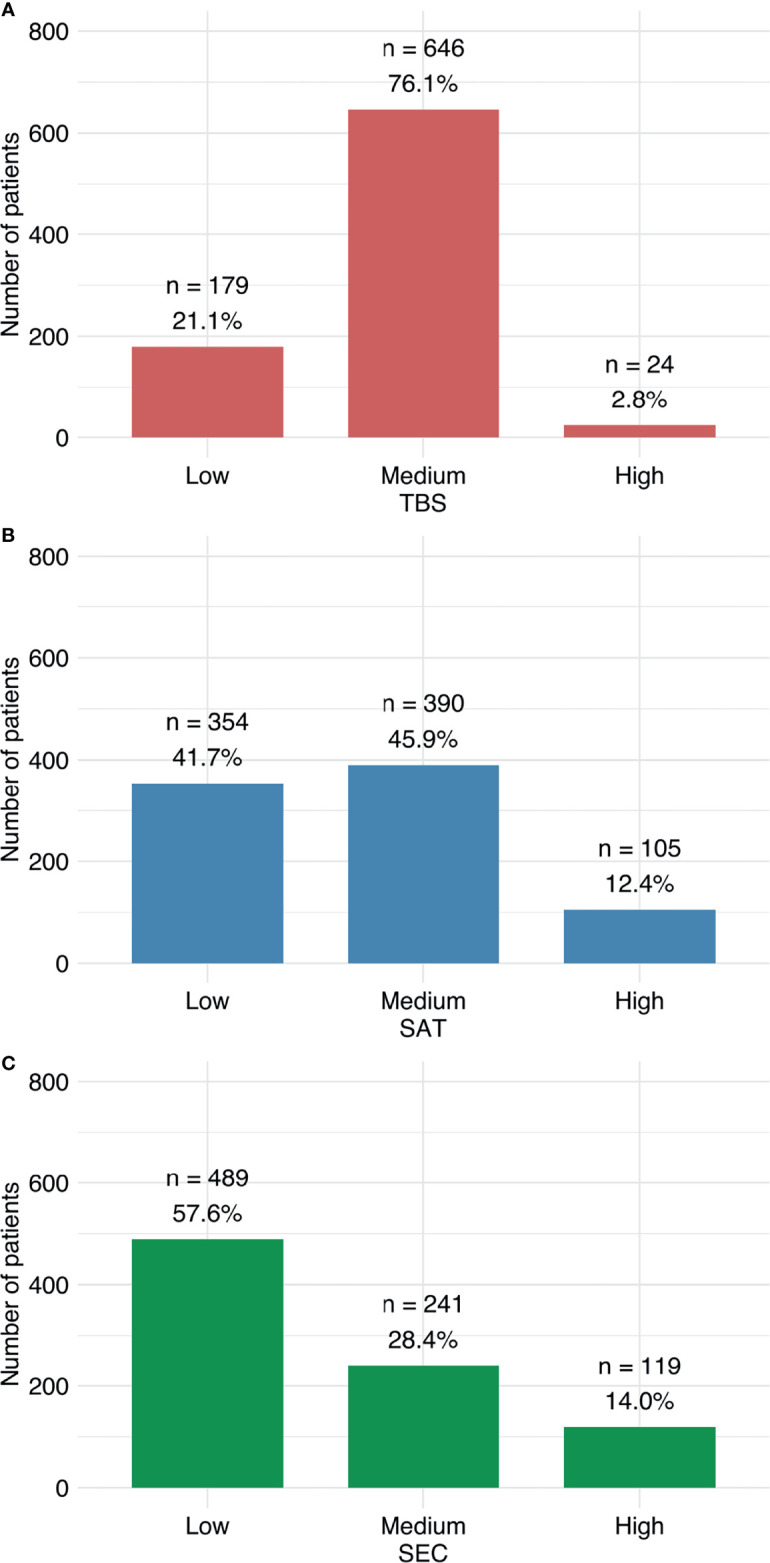
Distributions of patients among risk groups. Patients were classified as low, medium, or high risk, according to **(A)** TBS, **(B)** SAT, and **(C)** SEC scoring systems.

**Figure 1** illustrates the distributions of patients among different risk groups, according to the SAT, TBS, and SEC scoring systems. The distributions of patients among the risk groups were relatively balanced when applying the SAT and the SEC scores. However, the distribution was highly skewed, when the TBS score was applied; 76% of patients were classified as medium risk.

### Survival Analysis

**Figure 2:**
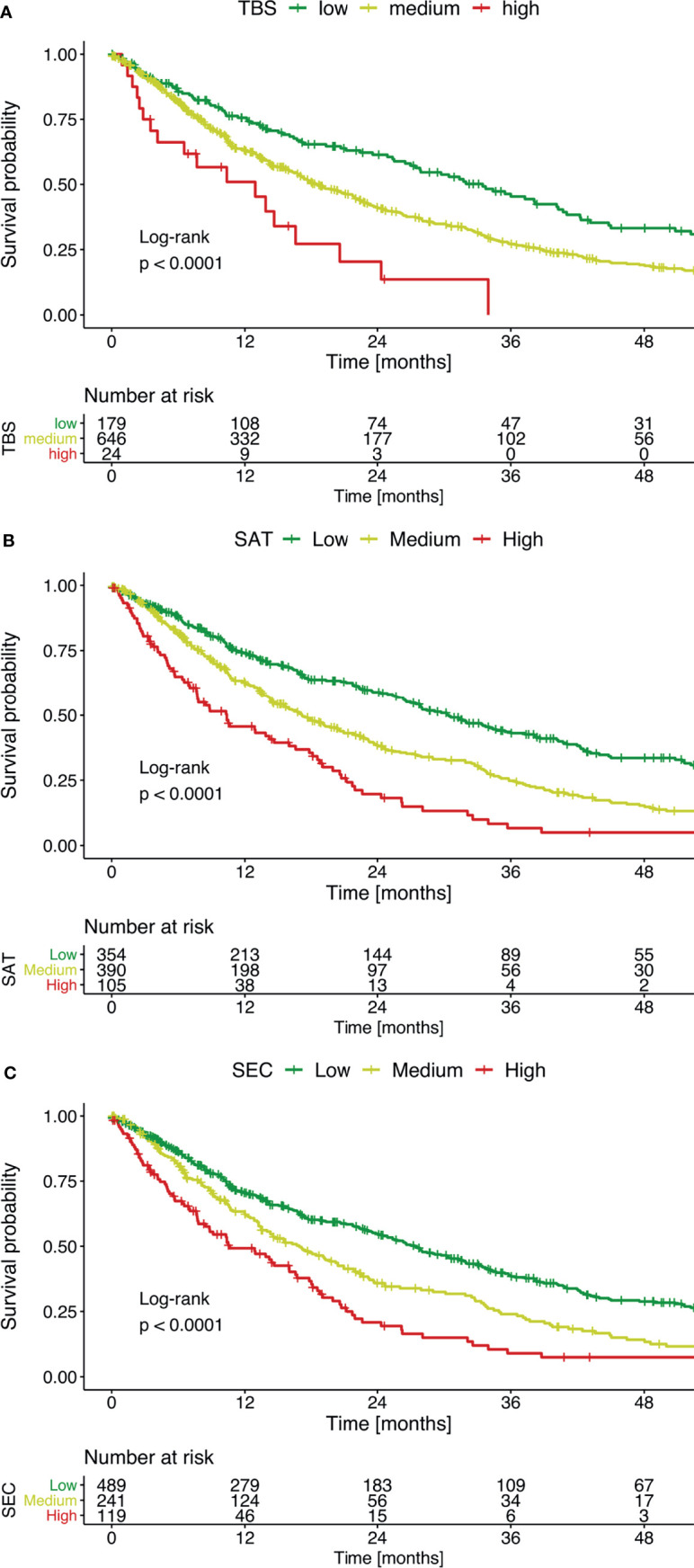
Kaplan-Meier curves show overall survival, stratified according to low, medium, or high mortality risk. Survival was evaluated separately with **(A)** the TBS, **(B)** the SAT, and **(C)** the SEC.

In univariate analyses of survival, all three scores showed significant differences between risk strata (**Figure 2** and **Table 3**). In the BCLC B subgroup, only the SAT and the SEC showed significant differences between risk strata, while the TBS reached no significance (**Supplementary Figure 1**). Notably, the distribution between the three risk groups was highly skewed in case of the TBS with 92.6% of the patients being in the intermediate group. Consequently, this score yielded no predictive power.

**Table 3:** Survival stratification, based on the risk of mortality, evaluated with the three different scores.

Score	Median OS (months)			p-value
	Low risk	Medium risk	High risk	
TBS	33.0	18.3	12.8	<0.001
SAT	30.0	16.9	10.2	<0.001
SEC	27.0	16.7	10.5	<0.001

TBS, tumor burden score. SAT, Six-and-Twelve score. SEC, Seven-Eleven criteria.

A univariate Cox hazard regression analysis indicated that all scores of tumor burden showed high prognostic value (**Table 4**). Among the other risk factors analyzed, the model showed that a low albumin level, a high bilirubin level, a high AST level, and a high INR were significant prognostic factors. In the subsequent multivariate analysis, despite a low albumin level and a high bilirubin level, only a SAT >6 and ≤12 remained an independent prognostic factor (**Table 4**). All other scoring systems lost their ability to predict the prognosis after TACE.

**Table 4:** Univariate and multivariate Cox proportional hazards regression model results for the influence of TBS, SAT, SEC, and other risk factors on the prognosis of TACE for patients with HCC .

Analysis		Univariate			Multivariate		
Covariate	Category	HR	95% CI	p-value	HR	95% CI	p-value
*Age*	*≥70 years*	0.9	0.7 – 1.1	0.110			
*AFP*	*>200 ng/ml*	1.0	0.8 – 1.2	0.970			
*Albumin level*	*<35 g/l*	2.2	1.8 – 2.6	**<0.001**	1.9	1.6 – 2.4	**<0.001**
*Bilirubin level*	*≥1.2 mg/dl*	1.9	1.6 – 2.2	**<0.001**	1.6	1.3 – 1.9	**<0.001**
*AST level*	*>31 U/l*	1.6	1.1 – 2.3	**0.005**	1.2	0.9 – 1.8	0.252
*ALT level*	*≥35 U/l*	1.1	0.9 – 1.3	0.470			
*INR level*	*>1.2*	1.5	1.2 – 1.8	**<0.001**	1.1	0.9 – 1.3	0.627
*TBS*	*Low*	Reference			Reference		
	*Medium*	1.5	1.2 – 1.9	**<0.001**	1.0	0.8 – 1.4	0.827
	*High*	2.9	1.9 – 5.1	**<0.001**	1.5	0.8 – 2.8	0.252
*SAT*	*≤6*	Reference			Reference		
	*>6 and ≤12*	1.7	1.4 – 2.0	**<0.001**	1.7	1.2 – 2.3	**0.003**
	*>12*	2.9	2.2 – 3.8	**<0.001**	2.1	0.8 – 5.2	0.113
*SEC*	*Low*	Reference			Reference		
	*Medium*	1.5	1.3 – 1.9	**<0.001**	0.9	0.7 – 1.2	0.618
	*High*	2.4	1.9 – 3.0	**<0.001**	1.1	0.5 – 2.5	0.827

AST, aspartate aminotransferase; ALT, alanine aminotransferase; AFP, alpha-fetoprotein; TBS, tumor burden score; SAT, Six-and-Twelve score; SEC, Seven-Eleven criteria.

P-values < 0.05 are depicted in bold.

In the BCLC B subgroup, the SAT score, the SEC score, albumin, bilirubin and INR reached significance in univariate analysis. Multivariate Cox hazard regression for these factors showed significance for the SAT score, albumin and bilirubin, whereas the SEC score and INR lost their predictive ability (**Supplementary Table 1**).

**Figure 3:**
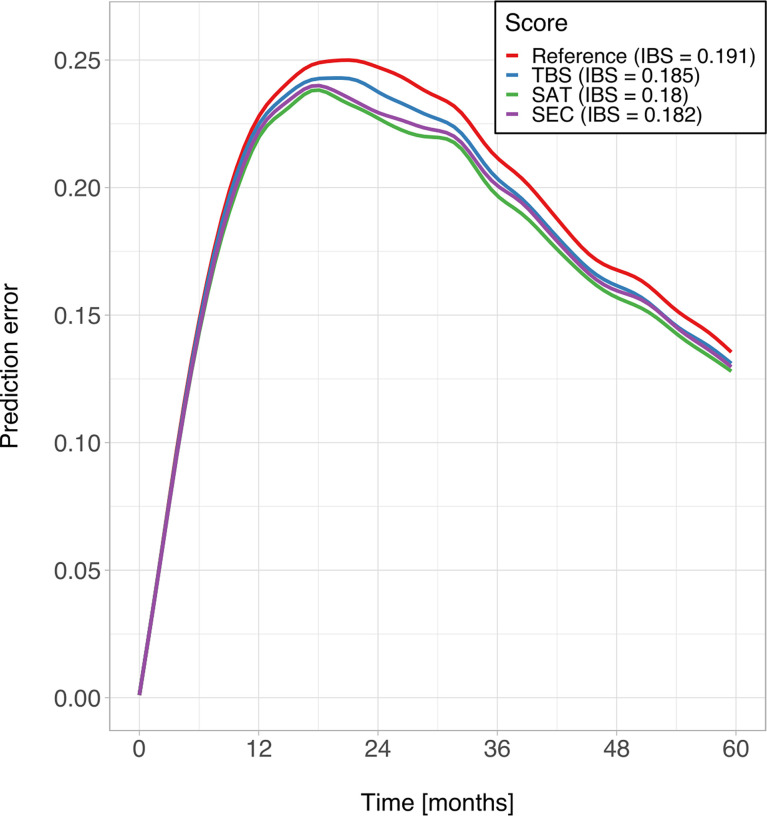
Prediction error curves for Kaplan-Meier estimates based on the TBS (blue), the SAT (green), the SEC (purple), compared to the unstratified sample (red).

The C-Indexes were close to 0.5 (not predictive) for all scoring systems. However, the SAT (0.59) had a slightly higher C-index than the TBS (0.54) and the SEC (0.58). The IBS values for the study interval [0 to 60 months] were 0.185 for the TBS, 0.180 for the SAT, and 0.182 for the SEC. Based on the Kaplan-Meier estimates of the unstratified sample, the reference IBS was 0.191. The prediction error curves, based on the Brier score, are shown in **Figure 3**. **Table 5** provides a detailed overview of the head-to-head comparison.

**Table 5:** Head-to-head comparisons of TBS, SAT, and SEC.

Scoring system	Category	Median OS	HR	95% CI		p-value	C-Index
**TBS**	**Low**	33.0	Reference				0.54
	**Medium**	18.3	1.5		1.2 – 1.9	**<0.001**	
	**High**	12.8	2.9		1.9 – 5.1	**<0.001**	
**SEC**	**Low**	27.0	Reference				0.58
	**Medium**	16.7	1.5		1.3 – 1.9	**<0.001**	
	**High**	10.5	2.4		1.9 – 3.0	**<0.001**	
**SAT**	**≤6**	30.0	Reference				0.59
	**>6 and ≤12**	16.9	1.7		1.4 – 2.0	**<0.001**	
	**>12**	10.2	2.9		2.2 – 3.8	**<0.001**	

Scoring systems are ordered according to their C-Indices. TBS, tumor burden score; SAT, Six-and-Twelve score; SEC, Seven-Eleven criteria.

P-values < 0.05 are depicted in bold.

## Discussion

To the best of our knowledge, this study was the first to perform a head-to-head-comparison of the SAT, the TBS, and the SEC for predicting the survival of patients with HCC undergoing TACE. In summary, all three scoring systems could successfully stratify patients in univariate analyses. However, only the SAT was identified as an independent prognostic factor in the multivariate analysis. Furthermore, all three scores showed only modest predictive ability.

In a recent study, Ho et al. promoted the TBS as a novel tumor burden-related scoring system and a new prognostic marker for patients with HCC undergoing TACE (19). The TBS was originally developed in patients with colorectal liver metastasis undergoing resections (34). Based on its promising results for those patients, several authors evaluated whether the TBS could be applied to patients with HCC undergoing curative treatments (35–37). However, among patients with HCC, the suitability for resection, according to the remnant liver function, differs tremendously between intermediate and advanced stages. For patients undergoing TACE, Ho et al. showed that the TBS could accurately discriminate risk stratifications for OS (19). However, subgroup analyses on patients within different BCLC stages and ALBI grades showed considerable overlap in the survival curves. Additionally, a comparison with stratification systems incorporating remnant liver function is missing.

Notably, in the Ho et al. study, the TBS showed a disparate distribution of patients in different risk categories. The TBS predicted that 72% of patient were at medium risk of mortality, compared to only 20% at low risk and 8% at high risk. Those findings were consistent with our results, where the TBS predicted that 76% were at medium risk, only 21% were at low risk, and only 3% were at high risk. This unequal distribution might have contributed to the lower predictive ability we found for the TBS compared to the SAT and the SEC, which showed a more balanced distribution (13, 20).

In contrast to our study cohort, viral liver disease was the most common etiology of HCC in the Ho et al. cohort (19). Furthermore, compared to that study, our cohort had considerably lower initial albumin levels and higher bilirubin levels, which indicated poor liver function. Similarly, in the initial reports on SAT and SEC, viral liver disease was the most common etiology, and liver function was considerably better in those cohorts than in our study cohort (13, 20). In the original study on the SAT, the score outperformed the BCLC classification and the ALBI score in predictive ability. In external validation studies, the C-Indexes for the SAT ranged between 0.60 and 0.70 (14–18). However, in all the validation studies, the predictive performance of the SAT was within the same range or somewhat below the performance of the BCLC and ALBI scoring systems (14). In the original study on the SEC, the authors did not compare the SEC with scoring systems that incorporated risk factors other than tumor burden (20). In our first external validation of the SEC score’s predictive performance, the C-Index was 0.58, which fell between the C-indexes of the SAT (0.59) and the TBS (0.54).

Our study showed that a low albumin level and a high bilirubin level were both independent predictors of impaired survival. Consequently, for our cohort, systems that took the remnant liver function into account performed best. These results confirmed our initial hypothesis that the complex interplay between cirrhosis and tumor development and progression might be underrepresented in scoring systems that are only based on tumor burden. Nevertheless, in daily clinical routine, a rapid estimation of tumor burden may provide a convenient bedside tool (particularly the SAT). Additionally, our results suggested the SAT performed better than the SEC and the TBS; thus, the SAT might be useful for characterizing study populations according to tumor burden in clinical trials. However, our results also indicated that none of the scores alone could support treatment decision making in daily clinical routines.

Apart from tumor burden and liver function, several other biomarkers play an important role in the evaluation of patients with HCC. Macrovascular tumor invasion has been identified as an independent factor in these patients (38, 39). Although macrovascular invasion appears more often in patients with higher tumor burden, it is not necessarily associated to multifocal tumor growth (40). Therefore, apart from the tumor burden, macrovascular invasion has to be considered as an additional and independent factor when evaluating biological tumor aggressiveness in these patients. Consequently, even in cases where tumor burden might be low, patients with macrovascular invasion are at higher risk for a poor post-TACE prognosis and immediate switch to systemic treatment should be discussed.

Following our results, we could not substantiate the benefit of continually developing novel scoring systems for ongoing scientific discussion. Instead, we recommend focusing on continually improving and updating the existing systems. Growing knowledge on novel risk factors, including the immune system and the nutritional status of patients, could be further integrated with growing knowledge of automated artificial intelligence-based risk predictions (25, 41). The novel developments in this field might allow integrating data pipelines in clinical and radiology information systems to facilitate the automation of highly individual risk predictions.

Clearly, the present study had several limitations. First, it had the limitations inherent to a retrospective study design. Thus, our results should be validated in a prospective trial. Second, we did not perform subgroup analyses of patients treated with different TACE techniques. However, in multiple previous studies, no significant differences in OS have been reported between conventional TACE and drug-eluting bead delivery TACE techniques (42–44). Moreover, recent study results have indicated that the applicability of various scoring and staging systems was similar for the different types of TACE (45).

## Conclusion

In this direct head-to-head comparison of three scoring systems for HCC prognosis, we found that the SAT was superior to the TBS and the SEC in survival stratification and in predictive ability. Thus, the SAT should be preferred when stratifying patients according to tumor burden; e.g., in the context of clinical trials. However, none of these scores was superior to established scoring systems in predicting survival. Therefore, they offered no added benefit for daily clinical routines, and treatment decisions should not be made based on tumor burden alone. Instead, a thorough interdisciplinary discussion that considers all types of risk factors is mandatory for improving patient stratification, and ultimately, patient survival.

## Data Availability Statement

The datasets presented in this article are not readily available because data cannot be shared publicly because of institutional and national data policy restrictions im-posed by the Ethics committee of the Medical Association of Rhineland Palatinate, Mainz, Germany since the data contain potentially identifying patient information. Data are available upon request for researchers who meet the criteria for access to confidential data. Requests to access the datasets should be directed to roman.kloeckner@unimedizin-mainz.de.

## Ethics Statement

The studies involving human participants were reviewed and approved by Ethics committee of the Medical Association of Rhineland Palatinate, Mainz, Germany. Written informed consent for participation was not required for this study in accordance with the national legislation and the institutional requirements.

## Author Contributions

LM, FH, TA, UF, BG, JH, SZ, M-SK, ME, TD, DB, VS, D-HC, DZ, DP, and RK devised the study, assisted in data collection, participated in the interpretation of the data, and helped draft the manuscript LM, TA, UF, JH, SZ, M-SK, ME, TD, DB, VS, DZ, and RK carried out the data collection. BG, D-HC, and DP supported the data collection efforts. LM, FH, DP, and RK created all of the figures and participated in the interpretation of data. LM, FH, DP, and RK performed the statistical analysis. All authors contributed to the article and approved the submitted version.

## Funding

LM is supported by the Clinician Scientist Fellowship “Else Kröner Research College: 2018_Kolleg.05”.

## Conflict of Interest

The authors declare that the research was conducted in the absence of any commercial or financial relationships that could be construed as a potential conflict of interest.

## Publisher’s Note

All claims expressed in this article are solely those of the authors and do not necessarily represent those of their affiliated organizations, or those of the publisher, the editors and the reviewers. Any product that may be evaluated in this article, or claim that may be made by its manufacturer, is not guaranteed or endorsed by the publisher.
