# Supplementary material for: Tumor Burden in Patients With Hepatocellular Carcinoma Undergoing Transarterial Chemoembolization: Head-to-Head Comparison of Current Scoring Systems
Source: Front Oncol. 2022 Feb 23;12:850454. doi: 10.3389/fonc.2022.850454 (PMC8904349; doi:10.3389/fonc.2022.850454)
Supplement: Supplementary file 1 [file DataSheet_1.docx]

**Supplementary Table 1:** Univariate and multivariate Cox proportional hazards regression model for the influence of TBS, SAT, SEC, and other established risk factors on the prognosis of patients with intermediate stage HCC undergoing TACE (BCLC B)

| **Analysis** | | **Univariate** | | | **Multivariate** | | | |
| --- | --- | --- | --- | --- | --- | --- | --- | --- |
| **Covariate** | **Category** | **HR** | **95% CI** | **p-value** | **HR** | **95% CI** | **p-value** | |
| *Age* | *≥70 years* | 1.0 | 0.7 – 1.3 | 0.800 |  |  |  | |
| *AFP* | *>200 ng/ml* | 1.2 | 0.9 – 1.5 | 0.300 |  |  |  | |
| *Albumin level* | *<35 g/l* | 2.1 | 1.6 – 2.7 | **<0.001** | 1.8 | 1.3 – 2.3 | **<0.001** | |
| *Bilirubin level* | *≥1.2 mg/dl* | 1.7 | 1.4 – 2.2 | **<0.001** | 1.5 | 1.1 – 1.9 | **0.006** | |
| *AST level* | *>31 U/l* | 1.3 | 0.8 – 2.1 | 0.270 |  |  |  | |
| *ALT level* | *≥35 U/l* | 1.0 | 0.8 – 1.3 | 0.950 |  |  |  | |
| *INR level* | *>1.2* | 1.6 | 1.2 – 2.1 | **<0.001** | 1.3 | 0.9 – 1.7 | 0.146 | |
| *TBS* | *Low* | Reference | | | | | | |
|  | *Medium* | 1.2 | 0.7 – 1.2 | 0.598 |  |  |  |  |
|  | *High* | 2.1 | 0.9 – 4.9 | 0.082 |  |  |  |  |
| *SAT* | *≤6* | Reference | | | | | |  |
|  | *>6 and ≤12* | 1.6 | 1.1 – 2.3 | **0.010** | 1.7 | 1.1 – 2.6 | **0.011** |  |
|  | *>12* | 2.7 | 1.8 – 4.1 | **<0.001** | 3.1 | 1.0 – 9.5 | **0.046** |  |
| *SEC* | *Low* | Reference | | | | | |  |
|  | *Medium* | 1.3 | 1.0 – 1.7 | **0.043** | 1.0 | 0.7 – 1.4 | 0.923 |  |
|  | *High* | 1.9 | 1.4 – 2.7 | **<0.001** | 0.9 | 0.3 – 2.4 | 0.768 |  |

**Supplementary Figure 1:** Kaplan-Meier curves show overall survival, stratified according to low, medium, or high mortality risk for the subgroup of patients within intermediate stage (BCLC B, n=418). Survival was evaluated separately with (A) the TBS, (B) the SAT, and (C) the SEC.

**

**Supplementary Figure 2:** Kaplan-Meier curves show overall survival, stratified according to low, medium, or high mortality risk for the subgroup of patients within intermediate stage (BCLC 0/A, n=284). Survival was evaluated separately with (A) the TBS, (B) the SAT, and (C) the SEC.

**Supplementary Figure 3:** Kaplan-Meier curves show overall survival, stratified according to low, medium, or high mortality risk for the subgroup of patients within intermediate stage (BCLC C/D, n=126). Survival was evaluated separately with (A) the TBS, (B) the SAT, and (C) the SEC.
